# The effects of nutritional habits and physical activity on treatment response and survival in patients with lung cancer

**DOI:** 10.1007/s00520-025-09409-6

**Published:** 2025-04-04

**Authors:** Zeynep Yılmaz Kaya, Deniz Kızılırmak, Yavuz Havlucu

**Affiliations:** Chest Diseases Department, Faculty of Medicine, Manisa Celal Bayar University, Manisa, Turkey

**Keywords:** Lung cancer, Mediterranean diet, Physical activity

## Abstract

**Background:**

Lung cancer is a malignancy marked by low treatment response rates and poor survival outcomes, despite significant advancements in diagnostic and therapeutic approaches. This study aims to examine the impact of dietary habits and physical activity levels on chemotherapy response and survival in patients diagnosed with advanced-stage non-small cell lung cancer.

**Methods:**

Patients diagnosed with stage IV non-small cell lung cancer and scheduled to receive platinum-based chemotherapy as the initial treatment were included in the study. Sociodemographic and cancer-related characteristics were documented. At the beginning of treatment, the patients’ dietary habits and physical activity levels were evaluated using the “Mediterranean Diet Adherence Scale,” the “International Physical Activity Questionnaire,” and the “Food Consumption Frequency Form,” while their average daily step count was calculated. The study investigated the relationships between dietary habits and physical activity levels with treatment response, 6-month survival, progression-free survival, and chemotherapy-related side effects.

**Results:**

The study included a total of 37 patients, 35 of whom were male, with a mean age of 63.49 years. The 6-month survival rate among these patients was 81.1%. In terms of treatment response, 35.1% of patients experienced disease progression, 54.1% demonstrated partial regression, and 8.1% achieved complete regression. Notably, the 6-month survival rate was significantly higher in minimally physically active patients compared to inactive patients (*p* = 0.022). Furthermore, patients adhering to the Mediterranean diet exhibited a significantly higher 6-month survival rate compared to those who did not follow the diet (*p* = 0.019). High adherence to the Mediterranean diet was also associated with a significantly lower incidence of chemotherapy-related gastrointestinal side effects (*p* < 0.001).

**Conclusion:**

This study demonstrated that physical activity and adherence to the Mediterranean diet positively impact 6-month survival and reduce the incidence of chemotherapy-related side effects.

## Introduction

Lung cancer, a malignancy originating from the bronchial epithelium, is responsible for approximately 1.6 million deaths globally each year [1]. At the time of diagnosis, nearly 47% of cases are in advanced stages [2]. Despite advances in treatment modalities, the 5-year survival rate for lung cancer patients remains just 15% [3].

Non-small cell lung cancer (NSCLC) constitutes 80–85% of all lung cancer cases [4]. For patients with metastatic NSCLC who have a good performance status but are not suitable candidates for targeted therapy or immunotherapy regimens, chemotherapy regimens are recommended. Combined chemotherapy regimens provide 1-year survival rates of approximately 30–40% in lung cancer patients [5].

Lung cancer is highly heterogeneous in terms of clinical presentation and response to treatment. In NSCLC cases, the stage of the disease is the most significant determinant of survival [6]. The treatment approach, usually determined based on the stage of the lung cancer and the patient’s performance status, directly influences survival outcomes [5]. In the past decade, an increasing number of independent factors have been proposed as determinants of survival in lung cancer. Since the early 1970s, diet has been extensively studied in relation to lung cancer risk, as dietary patterns may influence the elevated risk of lung cancer in smokers. Similarly, numerous studies have evaluated the relationship between physical activity and lung cancer. However, there is a limited body of research examining how dietary patterns and physical activity affect treatment response in patients with advanced lung cancer. This study aims to investigate the effects of nutritional habits and physical activity levels on 6-month survival, progression-free survival, treatment response, and treatment-related side effects in patients with metastatic NSCLC.

## Materials and methods

**Fig. 1:**
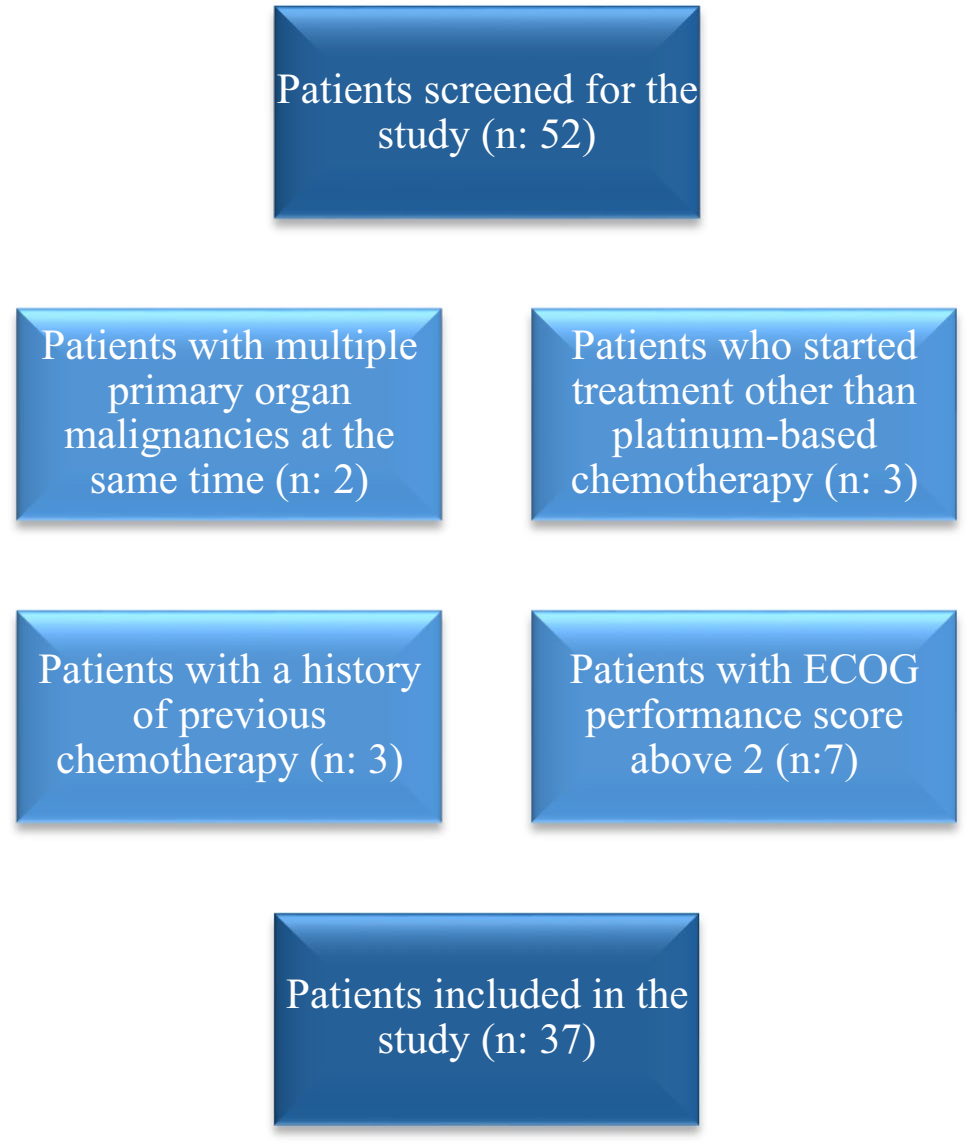
Consort diagram. n: number of patients, ECOG: Eastern Cooperative Oncology Group

This study was designed as a single-center, prospective cohort study. The study sample size was calculated as 44, based on an alpha value of 0.05, a power of 0.90, and an effect size of 0.5. All participants provided written informed consent in accordance with the Declaration of Helsinki and approved by the Manisa Celal Bayar University Faculty of Medicine Clinical Research Ethics Committee with decision number 370, dated January 2, 2023. Patients over the age of 18 who presented to the Pulmonology Clinic of the University Hospital between January 5 and July 5, 2023, and were newly diagnosed with stage 4 NSCLC with an Eastern Cooperative Oncology Group (ECOG) performance score of 2 or less were included in the study. Patients were excluded if they planned for treatments other than platinum-based chemotherapy as the initial treatment, were lost to follow-up, had a prior history of chemotherapy, or had multiple primary organ malignancies. After excluding 15 patients, a total of 37 participants, including 35 men, were enrolled in the study (Fig. 1).

The patients’ age, gender, height, weight, body mass index, educational level, occupational information, residential areas, comorbidities, pathological diagnoses, smoking histories, and ECOG performance scores were recorded. After assessment with positron emission tomography (PET-CT) and brain magnetic resonance imaging (MRI) at the initiation of treatment, the metastatic sites and the stages according to the 8th TNM staging system at the beginning of treatment were documented.

At the beginning of treatment, patients’ dietary habits and physical activity levels were evaluated using the “Mediterranean Diet Adherence Scale (MEDAS),” the “International Physical Activity Questionnaire,” and the “Food Consumption Frequency Form.” The patients’ MET-minutes per week scores were calculated, and their physical activity levels were classified as “inactive,” “minimally active,” and “highly active.” Additionally, patients were provided with pedometer devices to carry for 7 days to calculate their daily average step counts. The relationship between patients’ dietary habits and physical activity levels at the start of treatment, and their treatment responses, treatment-related side effect rates, as well as survival and progression-free survival at 6 months, was examined. Treatment response assessment at 6 months was performed using PET-CT. Response assessment was not performed for patients who received fewer than two cycles of chemotherapy or were unable to continue treatment due to death or severe side effects. In evaluating treatment response, the World Health Organization (WHO) criteria were used: complete response was defined as the complete disappearance of all disease sites; partial response was defined as a reduction of 50% or more in tumor size; stable disease was defined as a change in tumor size of less than 25% in either direction; and progressive disease was defined as an increase of more than 25% in tumor lesions and/or the appearance of new tumor sites. Chemotherapy-related side effects were categorized into hematological disorders, renal disorders, and gastrointestinal disorders. Adverse effects observed in patients over the 6-month period were monitored and recorded after each chemotherapy cycle.

### Mediterranean diet adherence scale

Martinez-Gonzalez et al. developed the Mediterranean Diet Adherence Scale in 2012. This scale is a 14-item survey that includes questions on various aspects of dietary habits, such as the type of essential oil used in meals, the daily consumption of olive oil, portions of fruits and vegetables, intake of margarine, butter, and red meat, weekly wine consumption, as well as the consumption of legumes, fish, seafood, snacks, nuts, pastries, and tomato sauce with olive oil. Additionally, it assesses whether white meat is preferred over red meat. The Mediterranean Diet Adherence Scale has been widely used in literature to assess diet compliance in patients with colon, breast, prostate, and lung cancer [7–9].

### International physical activity questionnaire

The International Physical Activity Questionnaire (IPAQ) is a standardized tool developed by researchers from various countries with support from the World Health Organization (WHO) and the Centers for Disease Control (CDC) to measure physical activity. Studies have demonstrated the validity of the IPAQ in cancer patients [10, 11]. The IPAQ has been successfully administered in previous studies involving cancer patients [12].

### Statistical evaluation

The data obtained in the study were statistically analyzed using “SPSS Statistics 21.” Descriptive statistics, including frequency, percentage values, median (interquartile range), mean and standard deviation were determined. For comparisons, independent sample *t*-tests (Student’s *t*-test) and one-way analysis of variance (ANOVA) were used for normally distributed numerical variables, while the Mann–Whitney *U* test was employed for numerical variables that did not follow a normal distribution. Comparisons between categorical variables were performed using the chi-square test. Survival curves and probabilities were calculated using the Kaplan–Meier technique. Comparative correlation analyses (Pearson) were applied to identify variables affecting mortality. A *p*-value of < 0.05 was considered statistically significant.

## Results

Among the 37 patients included in the study, 35 (94.5%) were male, with an average age of 63.49 years. Comorbid conditions were present in 21 patients (56.8%). The sociodemographic characteristics of patients are presented in Table 1.

**Table 1:** Sociodemographic characteristics

Age (years), mean ± SD	63.49 ± 7.52
Gender (F/M), *n* (%)	35 (94.6) / 2 (5.4)
Height (cm), mean ± SD	168.95 ± 7.59
Weight (kg), mean ± SD	66.95 ± 14.58
Body mass index, *n* (%)	
Underweight	2 (5.4)
Normal	25 (67.6)
Overweight	6 (16.2)
Obese	6 (16.2)
Residential area, *n* (%)	
Urban	21 (56.8)
Rural	16 (43.2)
Educational status, *n* (%)	
Illiterate	4 (10.8)
Literate	1 (2.7)
Primary education	29 (78.4)
Secondary education	3 (8.1)
Occupation, *n* (%)	
Housewife	1 (2.7)
Worker	11 (29.7)
Farmer	17 (45.9)
Tradesman	1 (2.7)
Other	7 (18.9)
Smoking status, *n* (%)	
Smoker	11 (29.7)
Former smoker	25 (67.6)
Non-smoker	1 (2.7)
Smoking (pack-year), mean ± SD	50.03 ± 25.207
Comorbid diseases, *n* (%)	
Present comorbid disease	21 (56.8)
Hypertension	8 (21.6)
Diabetes mellitus	7 (18.9)
COPD	14 (37.8)
Coronary artery disease	11 (29.7)

*SD*, standard deviation; *n*, number of patients; *%*, percentage rate among all patients; *COPD*, chronic obstructive pulmonary disease

Thirteen patients (35.1%) were diagnosed with adenocarcinoma, 17 patients (45.9%) with squamous cell carcinoma, and 7 patients (18.9%) with not otherwise specified (NOS) NSCLC. Cancer-related characteristics are summarized in Table 2.

**Table 2:** Cancer-related features

Pathological diagnosis, *n* (%)	
Adenocarcinoma	13 (35.1)
Squamous cell carcinoma	17 (45.9)
NSCLC, NOS	7 (18.9)
Disease stage, *n* (%)	
4A	22 (59.5)
4B	15 (40.5)
T, *n* (%)	
T1	2 (5.4)
T2	10 (27)
T3	10 (27)
T4	15 (40.5)
*N*, *n* (%)	
N0	4 (10.8)
N1	2 (5.4)
N2	20 (54.1)
N3	11 (29.7)
*M*, *n* (%)	
M1a	14 (37.8)
M1b	9 (24.3)
M1c	14 (37.8)
Metastasis sites, *n* (%)	
Contralateral lung	17 (45.9)
Liver	10 (27)
Bone	11 (29.7)
Surrenal	7 (18.9)
Brain	5 (13.5)
Performance status, *n* (%)	
ECOG 0	18 (48.6)
ECOG 1	15 (40.5)
ECOG 2	4 (10.8)

*n*, number of patients; *%*, percentage among all patients; *NSCLC*, non-small cell lung cancer; *NOS*, not otherwise specified; *T*, primary tumor; *N*, regional lymph nodes; *M*, distant metastasis; *ECOG*, Eastern Cooperative Oncology Group

Seventeen patients (45.9%) were non-compliant with the Mediterranean diet. Twenty patients adhered to the Mediterranean diet, with 11 patients (29.7%) demonstrating acceptable compliance and 9 patients (24.3%) showing strict compliance. Based on the MET-min/week score, 23 patients (62.2%) were classified in the inactive group, while 14 patients (37.8%) were in the minimally active group. The average daily step count of the patients was calculated as 1495.75 ± 1473.76 (Table 3).

**Table 3:** Physical activity, diet, and healthy lifestyle scores

Mediterranean diet adherence	
Lowest adherence to the MD	17 (45.9)
Highest adherence to the MD	20 (54.1)
MET-minutes per week, mean ± SD	527.01 ± 463.77
Physical activity level, *n* (%)	
Inactive	23 (62.2)
Minimally active	14 (37.8)
Average number of steps per day	1495.75 ± 1473.76

*n*, number of patients; *%*, percentage among all patients; *MD*, Mediterranean diet; *SD*, standard deviation; *MET*, metabolic equivalent (metabolic value)

**Fig. 2:**
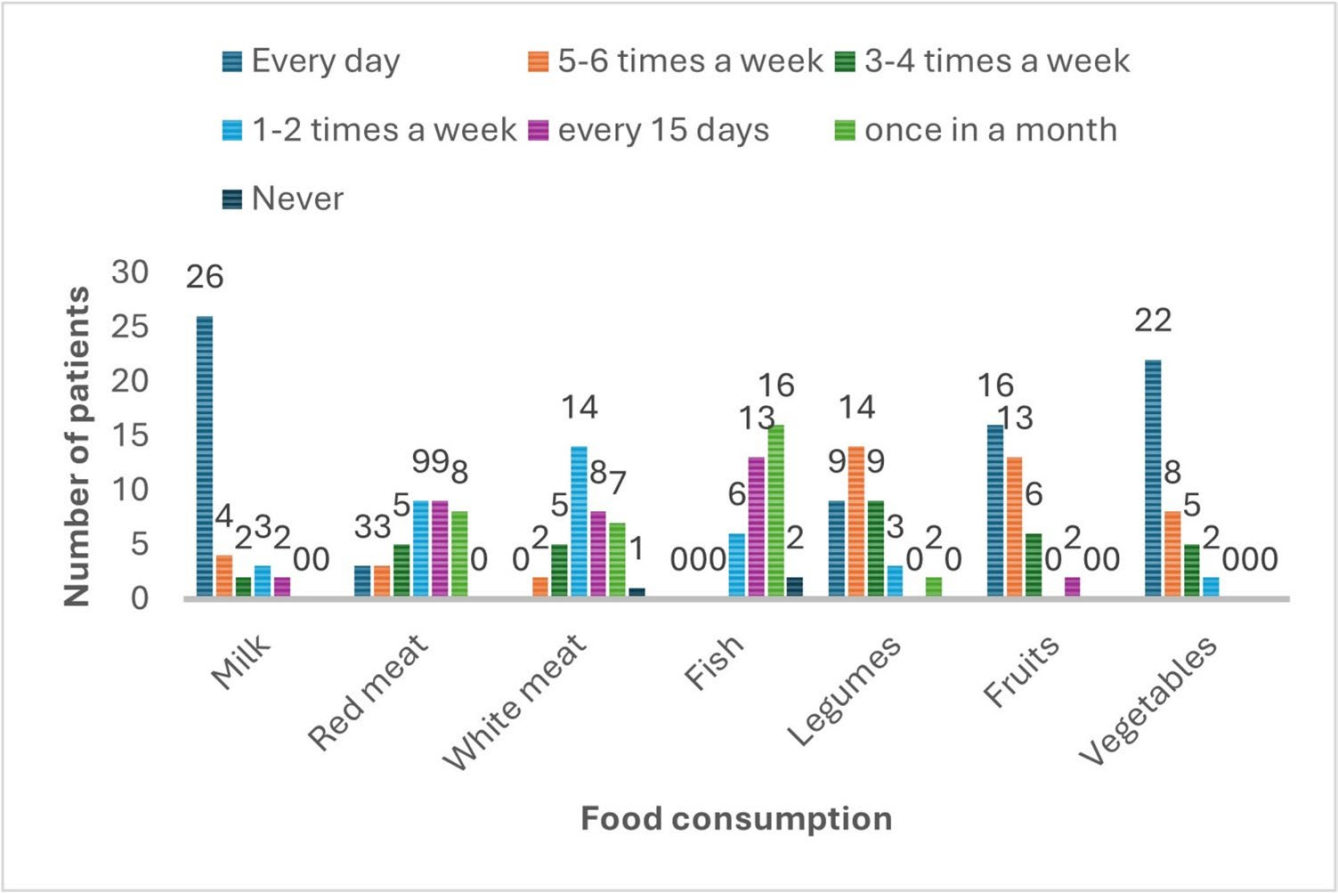
Food consumption frequency

In the patients included in the study, a high frequency of consumption of dairy products, fruits, vegetables, and legumes was observed, while the consumption frequency of red meat, white meat, and fish was lower (Fig. 2).

At the conclusion of the designated follow-up period, the 6-month survival rate of the patients was calculated to be 81.1%. No statistically significant relationships were found between the patients’ age (*p* = 0.530), body mass index (*p* = 0.165), or smoking pack-years (*p* = 0.21) and their 6-month survival. Additionally, no significant associations were observed between the patients’ pathological diagnoses and their 6-month survival (*p* = 0.053) or progression-free survival (*p* = 0.489). However, disease stage and ECOG performance status were found to be associated with both 6-month survival and progression-free survival.

**Fig. 3:**
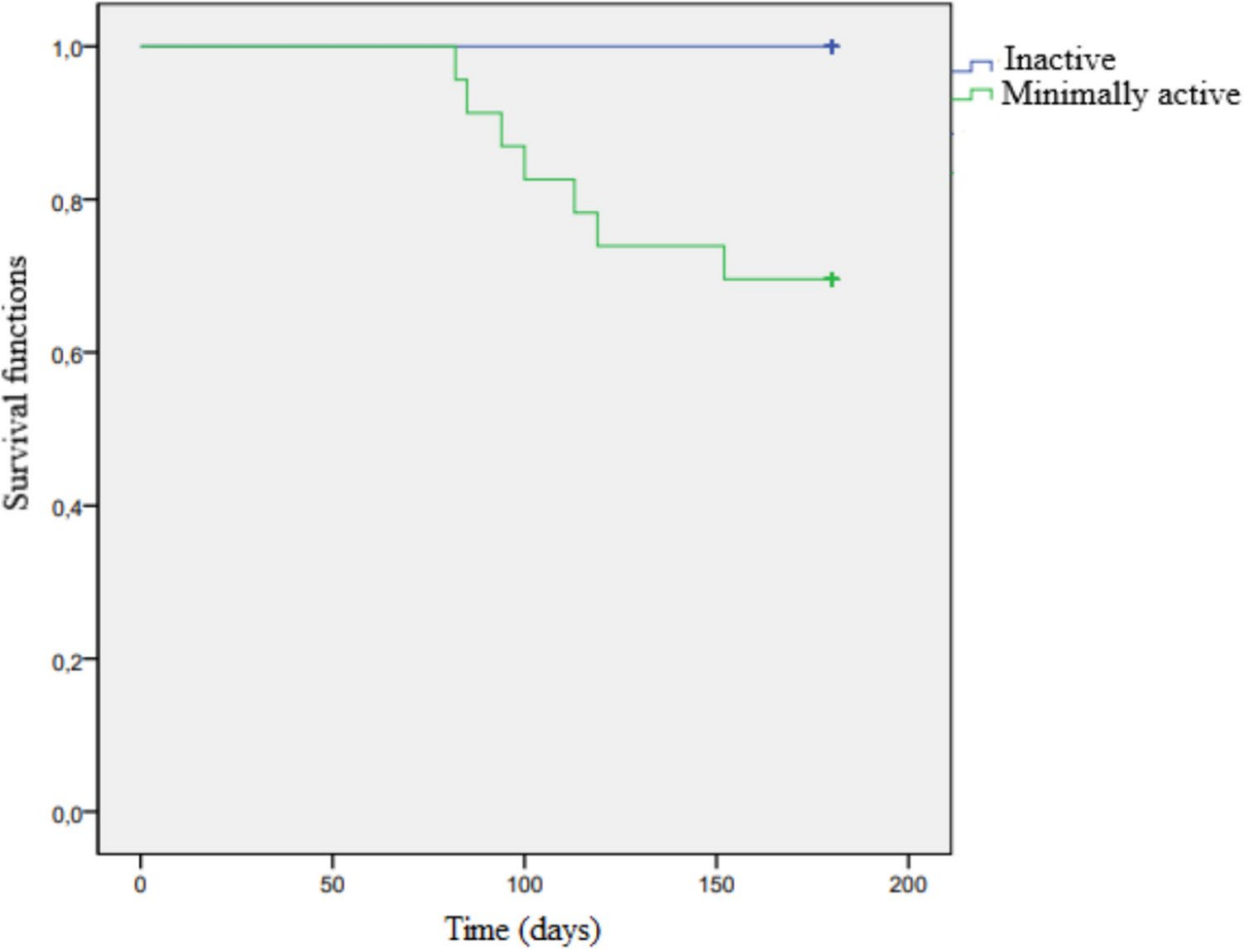
Association between physical activity and 6-month survival

The 6-month survival rate was statistically significantly higher in physically minimally active patients compared to inactive patients (*p* = 0.031) (Table 4). Survival analysis using the Kaplan–Meier method revealed that the 6-month survival probability was higher in physically minimally active patients than in inactive patients (*p* = 0.026) (Fig. 3). Among the patients who survived at the end of six months, both the MET-min/week score (*p* = 0.009) and the average daily step count (*p* = 0.024) were found to be significantly higher.

**Table 4:** Effect of physical activity level and Mediterranean diet compliance on 6-month survival

		Physical activity level, *n* (%)		*p* value	Mediterranean diet adherence, *n* (%)		*p* value
		Inactive	Minimally active		Lowest adherence to the MD	Highest adherence to the MD	
6-month survival	Surviving	16 (43.24)	14 (37.83)	0.031*	11 (29.72)	19 (51.35)	0.033*
	Deceased	7 (18.91)	0 (0)		6 (16.21)	1 (2.7)	

*n*, number of patients; *%*, percentage among all patients; *statistically significant

**Fig. 4:**
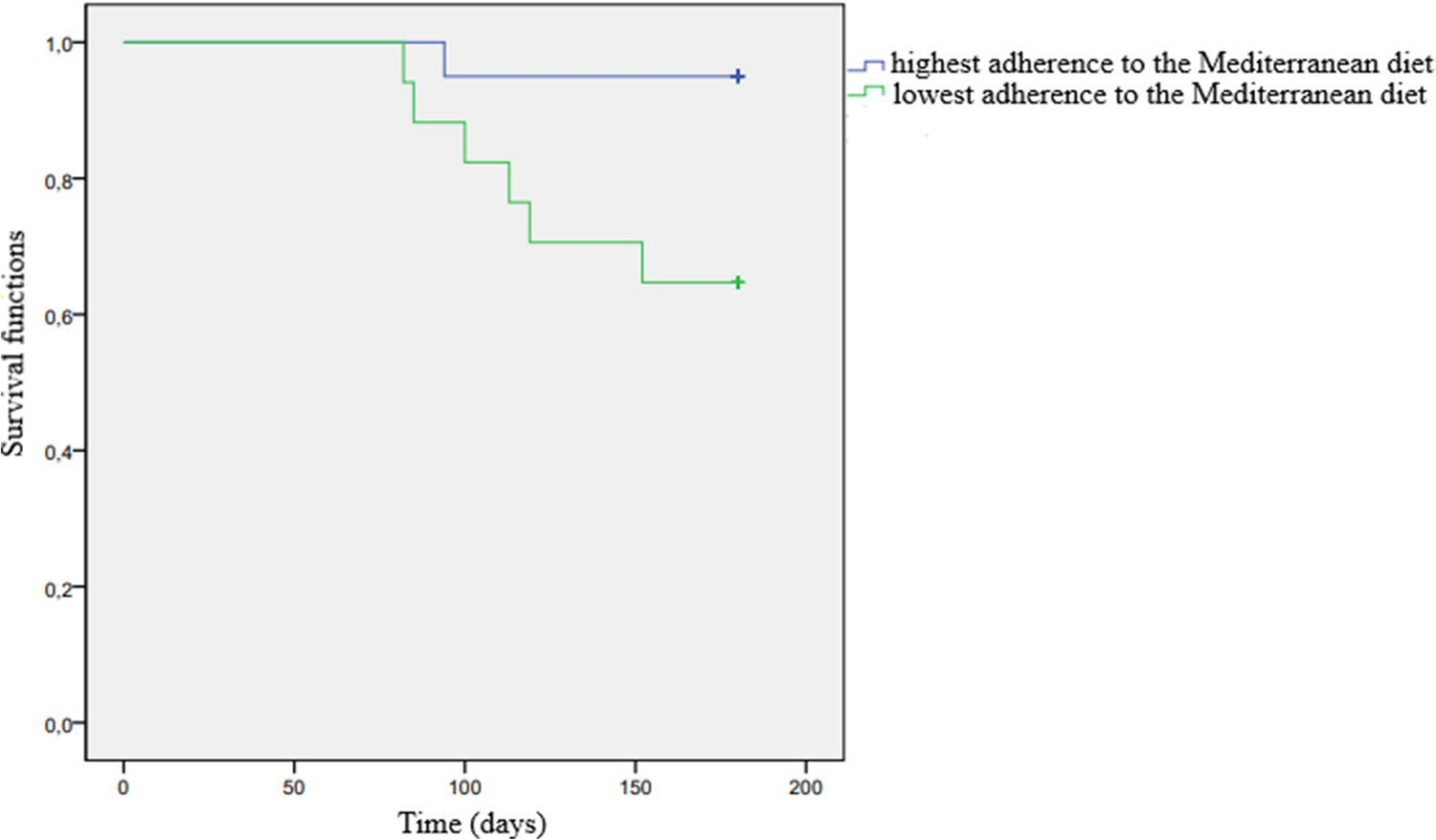
Association between Mediterranean diet adherence and 6-month survival

No significant association was found between the frequency of food consumption and 6-month survival or progression-free survival. However, patients who adhered to the Mediterranean diet had a significantly higher 6-month survival rate compared to those who did not adhere to the diet (*p* = 0.033) (Table 4). Survival analysis using the Kaplan–Meier method showed that the 6-month survival probability was significantly higher in patients who adhered to the Mediterranean diet compared to those who did not (*p* = 0.021) (Fig. 4).

Regarding treatment responses, 35.1% of patients experienced progression, 54.1% had partial regression, and 8.1% achieved complete regression. The disease remained stable in 1 patient (2.7%). No significant relationship was found between pathological diagnosis and treatment responses (*p* = 0.660). Treatment responses were statistically significantly higher in stage 4A patients compared to stage 4B patients (*p* = 0.024). Patients with an ECOG performance score of 0 had significantly higher treatment responses (*p* = 0.019). No significant relationship was observed between physical activity level (*p* = 0.346) or adherence to the Mediterranean diet (*p* = 0.169) and treatment responses.

During the treatment period, 32 patients (86.5%) developed chemotherapy-related side effects. Hematological side effects were observed in 22 patients (59.5%), nephrological side effects in 9 patients (24.3%), and gastrointestinal system side effects in 23 patients (62.2%). The overall rate of chemotherapy-related side effects was significantly higher in physically inactive patients (*p* = 0.005). Patients with high adherence to the Mediterranean diet had a significantly lower rate of overall chemotherapy-related side effects (*p* = 0.022) and a significantly lower incidence of gastrointestinal system side effects related to chemotherapy (*p* < 0.001).

## Discussion

In our study, adherence to the Mediterranean diet and higher levels of physical activity positively influenced overall survival; however, they were not associated with progression-free survival or treatment response. Improved treatment responses were observed in patients with higher ECOG performance scores and those classified as stage 4A at the start of treatment. Patients adhering to the Mediterranean diet and engaging in higher levels of physical activity experienced a lower likelihood of developing general chemotherapy-related side effects. Furthermore, patients following the Mediterranean diet demonstrated a reduced probability of gastrointestinal side effects.

To date, nutritional strategies that may positively impact cancer management have been extensively studied; however, definitive findings remain scarce. A prospective analysis conducted by Jansen et al. over more than 25 years demonstrated an inverse relationship between fruit consumption and lung cancer mortality [13]. Additionally, a study by Murphy et al. involving lung cancer patients undergoing chemotherapy observed higher response rates and one-year survival among those using fish oil compared to those receiving standard care. It has been suggested that fish oil supplementation may enhance chemotherapy efficacy and survival without affecting the side effect profile [14]. In our study, no significant association was found between the frequency of consumption of dairy products, fruits, vegetables, legumes, white meat, fish, and red meat and 6-month survival, progression-free survival, treatment response, or chemotherapy-related side effects. However, due to the study design, dietary intake frequencies were assessed at the start of treatment. Changes in food intake frequencies during the 6-month period could have occurred due to side effects such as loss of appetite, nausea, and taste alterations during chemotherapy. This limitation has made it impossible to observe the impact of dietary intake frequencies during the treatment process.

There are approaches suggesting that focusing on dietary patterns rather than individual nutrients might provide a more comprehensive understanding of the interactions between foods and clarify the connections between diet and cancer. The Mediterranean diet is characterized by high consumption of plant-based foods, particularly whole grains, vegetables, fruits, nuts, and legumes, along with regular intake of fish and seafood, and low consumption of eggs, red and processed meats, and high-fat dairy products. The beneficial effects of the Mediterranean diet are attributed to various bioactive compounds, such as polyphenols, monounsaturated and polyunsaturated fatty acids, and fiber. These compounds are linked to the reduction of blood lipids, protection against oxidative damage, improvement of insulin sensitivity, enhancement of endothelial function, and promotion of antithrombotic activity [15]. The positive association between the Mediterranean diet and cancer is primarily due to the high content of antioxidants and anti-inflammatory nutrients found in many Mediterranean foods (e.g., legumes, fresh fruits, nuts, vegetables, fish, and olive oil, particularly extra virgin olive oil). These nutrients have a protective effect against cellular degeneration and the proliferation of cancer cells. Additionally, the high concentration of polyphenols in olive oil, wine, and vegetables plays a crucial role in reducing cancer cell proliferation and protecting cell membranes from metastasis [16, 17]. Studies have associated high adherence to the Mediterranean diet with a 15% reduced risk of lung cancer among former smokers [18]. Another study involving 6370 individuals who were asked about their diets after cancer diagnosis revealed that adherence to the Mediterranean diet was associated with a 26% reduction in all-cause mortality and a 29% reduction in cancer-specific mortality [19]. In our study, the 6-month survival rate was found to be 29.72% in patients not adhering to the Mediterranean diet, compared to 51.35% in those with acceptable or strict adherence to the diet. Even with a small sample size, this apparent positive effect suggests that adherence to the Mediterranean diet may significantly enhance cancer-related survival. Some meta-analyses have indicated that dietary patterns, including the Mediterranean diet, could mitigate chemotherapy-related nausea and vomiting [20]. Our study supports these findings by demonstrating that patients adhering to the Mediterranean diet experienced statistically significantly fewer gastrointestinal side effects.

In recent years, the number of studies providing evidence-based data on physical activity to reduce mortality in cancer patients has increased. A meta-analysis by Friedenreich et al. found that higher physical activity was associated with a statistically significant 19% reduction in lung cancer mortality [21]. However, another study involving 111 lung cancer patients reported that a 2-month exercise program designed to increase physical activity by 3 MET-hours per week had no effect on overall mortality [22]. The findings of our study indicate that the 6-month survival rate was significantly higher in patients who engaged in minimal physical activity compared to those who were inactive. The observation that 62.2% of the patients included in the study were classified as inactive, with a low average daily step count, indicates that advanced-stage lung cancer patients tend to exhibit low levels of physical activity. This situation may be related to the patients’ advanced age, a history of smoking, and the presence of comorbidities such as COPD, which can cause respiratory symptoms and limit physical performance, particularly exertional dyspnea. Indeed, 37.8% of the patients included in the study had a diagnosis of COPD in addition to lung cancer.

The primary limitations of our study include its single-center design and the lack of long-term follow-up data. Furthermore, due to the limited number of patients available for multivariate analysis, factors influencing 6-month survival, progression-free survival, treatment response, and side effects could only be evaluated using univariate analysis. Additionally, since 94.5% of the participants were male, our findings are limited in their ability to assess gender-based differences. While the patients’ dietary habits were evaluated at the onset of treatment, changes in these habits during chemotherapy, induced by side effects, were not monitored. Therefore, further long-term studies are necessary to investigate how dietary habits evolve throughout treatment and how these changes may influence treatment outcomes.

Another limitation of the study is that patients’ dietary habits were recorded through self-reports, and their dietary habits were not objectively observed. Similarly, while the questionnaire used to assess physical activity was also self-reported, objective data on physical activity levels were obtained by measuring daily step counts using a pedometer.

Moreover, the study did not account for socioeconomic factors, which could potentially influence cancer treatment outcomes and patients’ lifestyle habits. Socioeconomic status has been shown to impact both the accessibility to healthcare and the adoption of healthy lifestyle choices, such as diet and physical activity. The exclusion of these factors from our analysis may limit the generalizability of our findings. Future research should consider incorporating socioeconomic factors to better understand their role in influencing treatment outcomes and lifestyle behaviors.

The notable strengths of our study include its design as a prospective cohort study and the close monitoring of patients over a 6-month period to minimize data loss. By creating a homogeneous patient cohort, we aimed to exclude confounding factors as much as possible. Additionally, evaluations were conducted using widely accepted objective measurements and scales, providing valuable data on the follow-up and prognosis of advanced-stage lung cancer patients, a population with limited representation in the literature.

## Conclusions

Increasing physical activity levels, dietary planning, and interventions aimed at improving quality of life may positively contribute to the treatment process for patients diagnosed with lung cancer. The Mediterranean diet can be safely recommended for lung cancer patients undergoing chemotherapy, especially those experiencing significant gastrointestinal symptoms. Larger, multicenter studies are needed to further investigate the effects of physical activity and dietary patterns on outcomes in lung cancer patients.

## Data Availability

No datasets were generated or analysed during the current study.
